# Computational Analyses of Stepped-Lap Composite Repairs on a Full-Scale Wing Model

**DOI:** 10.3390/polym18050570

**Published:** 2026-02-26

**Authors:** Alihan Cambaz, Huseyin Enes Salman

**Affiliations:** Department of Mechanical Engineering, TOBB University of Economics and Technology, Ankara 06560, Türkiye; a.cambaz@etu.edu.tr

**Keywords:** composite repair, adhesively bonded stepped-lap, on-site repair, Hashin criterion, finite element model

## Abstract

The use of carbon fiber-reinforced plastic (CFRP) components has increased significantly in civilian aviation, necessitating effective maintenance and repair strategies to ensure durability and performance. While prior studies have focused on composite repair methods, such as stepped scarf patch and bolted joint repairs, these were limited to specimen and panel levels without addressing full-scale wing models. This study bridges that gap by evaluating stepped-lap repairs on a full-scale composite wing model under realistic loading conditions and exploring various repair scenarios. To reduce computational cost, two-dimensional shell elements were employed to simulate repairs, with results validated using experimental tensile test data from stepped-lap repaired specimens. Numerical models were developed for single regions and two closely located repair regions. For single-region repairs, adding up to two extra layers enhanced mechanical strength, but three extra layers increased strain, diminishing performance. For two closely located repairs, additional layers improved strength, though less effectively than single-region repairs. Square-shaped repairs exhibited higher strain due to stress concentrations at the corners, while circular repairs showed more uniform stress and strain distribution. These findings emphasize the importance of optimizing repair geometry and layer configurations using numerical simulations to ensure optimal structural performance of CFRP components.

## 1. Introduction

The increase in carbon-fiber reinforced plastic (CFRP) components has risen significantly as a primary structure both in civilian aviation, such as in the Boeing 787 and A350, and in fifth-generation fighter aircraft, such as in the F-35 and F-22, compared to conventional metal structures [1,2]. With the increasing use of CFRP in primary load-bearing aerospace structures, there is a rising demand in the industry for composite materials that offer high specific strength, specific stiffness, extended fatigue life, and durable repair procedures [3,4]. Despite their advantages, CFRP materials are prone to internal damage, including debonding, wrinkles, delamination, low-velocity impact, and low interlaminar toughness [5,6]. To maintain structural integrity, in-service damage exceeding the limits of established structural repair procedures must be addressed within the quality assurance schedule, in compliance with airworthiness regulations [7,8].

Structural repair is a key necessity in the aerospace industry, considering the new adhesive-bonded repair techniques for maintaining structural integrity, ensuring the feasibility of repair, achieving uniform stress distribution compared to bolted joints, and ensuring cost-effectiveness instead of structure replacement [9,10,11]. The two most common methods for joining a parent structure to a repair structure, superior to bolted joints, butt strap joints, and overlap joints, are stepped-lap and tapered scarf joints, which transfer loads primarily through shear in the adhesive [11,12,13,14]. Adhesively bonded joints perform better in distributing loads, eliminating high stress concentration around fastener holes and extra undue weight, and ensuring galvanic compatibility [13,15,16]. Nevertheless, the aerospace industry currently lacks a standardized procedure for designing bonded repair joints, which becomes even more challenging for scarf or stepped repairs due to the variable stiffness along the bondline caused by ply drop-off in the adherends [4,9].

In stepped-lap bonded repairs, material removal is mainly done using conventional machining, such as milling with a hand-held router by an authorized skilled technician [4,17]. Although non-conventional machining techniques such as wet/dry laser techniques have been recently developed as a new automated process, the removal rate of material is lower than conventional milling methods, and heating the material leads to heat-affected zones that may reduce the residual strength [18,19]. In bonded repair patch manufacturing, there are two basic options: hard (dry) and soft (wet) [18,20]. While the hard patch is a procured patch that is secondarily bonded to the parent structure using adhesive film, the soft patch configuration involves wet plies that are placed in the repair region and co-bonded to the parent structure [18,21]. Co-bonded curing structures eliminate many issues, such as poor patch consolidation, variation in bondline thickness, patch wrinkles, and voids, which can significantly reduce the interlaminar shear and bending strength of laminates compared to secondary bonded curing [22,23].

In the current situation, the aerospace industry does not have a standard procedure for designing bonded repair joints, and the issue becomes considerably more complex when applying stepped repairs because of the varying stiffness that causes the load path to shift as a result of plies dropping off [24]. For this reason, in recent years, various modeling approaches have been investigated to design composite bonded repairs to determine the ultimate strain and stress at failure [25,26,27]. The majority of research focuses on analyzing equivalent joints, using two-dimensional (2D) shell elements and three-dimensional (3D) solid elements to represent real-world stepped-lap repairs and capture all relevant effects [28,29].

In a simplified 2D model at the specimen level, it is assumed that all the load is transmitted through the adhesive bond, disregarding the bypass loads that are not directly transferred through the adhesive bond in the repaired area [11]. Few studies have taken into account the 3D model of the repaired parts, and these numerical modeling and test studies have been at the specimen level and rarely at the panel level [11,17,20,30]. However, shear locking is the primary issue when modeling thin-walled composite plies using 3D solid linear elements such as C3D8 [15]. Shear locking is an overly stiff behavior and is unable to capture kinematic deformation under bending conditions [31]. When solid parts have one dimension significantly smaller than the other two, the locking phenomenon, which introduces artificial stress, becomes considerably more pronounced [32]. In order to precisely circumvent this problem, three or four parts need to be distributed uniformly across the thickness [33]. This mesh configuration will capture the effects of stiffness and bending [34]. Nevertheless, this method generates an enormous number of degrees of freedom for the repair scenarios.

This paper aims to reduce computational demand by investigating various stepped-lap repair scenarios on a full-scale aircraft wing, using 2D shell elements to model the repairs. A partition face was utilized to model the stepped-lap repair on a single shell plane, and the approach was validated experimentally at the specimen level using the ASTM 3039D uniaxial tension test [34]. This study addresses several novel contributions: (1) Unlike many studies, it examines a full-scale wing model including spar, rib, and sandwich panel skin designed according to industry standards and repaired using the stepped-lap method; (2) it investigates the effects of multiple stepped-lap repairs and their geometric configurations; (3) it examines the impact of damaged plies on joint performance; and (4) it studies the behavior of wet-method repairs on structures made with prepreg material. Focusing on stepped repairs, which involve machining plies into a stepped shape, this paper addresses the suitability of industrial wet lay-up repairs, as they more closely align with stepped-lap configurations than scarf configurations due to ply drop-off. The repair patches are thus manufactured using the wet−soft method and directly cured via co-bonding.

## 2. Materials and Methods

In this study, a full-scale wing model including spars, structural ribs, anti-sloshing ribs, trailing edge ribs, and skin was created to model various repair scenarios on the sandwich panel skin. The damage and associated repairs on the panel skin are virtually generated and modeled using ABAQUS 6.14 Standard software. Due to the large geometry of the full-scale wing model, 2D shell elements are used in the models to reduce the computational cost. To verify the accuracy of results when using shell elements, tensile testing of stepped-lap repaired specimens was initially modeled in ABAQUS, and tensile tests were conducted in compliance with ASTM 3039D to validate the numerical results of the simulations.

To apply realistic boundary conditions to the full-scale wing models, aerodynamic forces acting on the panel skin were determined through computational fluid dynamics (CFD) simulations performed in ANSYS Fluent [35] In addition to aerodynamic forces, the weights of the ammunition units and fuel were included in the full-scale wing models. The boundary conditions in the finite element (FE) modeling were designed to closely replicate real-world conditions. Furthermore, the 2D Hashin criterion was implemented in ABAQUS using the UMAT subroutine with user-defined material parameters.

### 2.1. Experimental Validation of the Shell Method

To demonstrate the accuracy of shell elements, an experimental study was first conducted to validate the applied numerical solution method. For this purpose, tensile tests were performed on composite specimens, and the failure modes observed in these tests were recorded. These experimental failure modes were then compared with those predicted by the numerical models.

Full-scale composite wing structure repair assessments are derived from investigations of mesoscale composite repair specimens. In compliance with ASTM 3039D guidelines, medium-scale composite specimens measuring 25 mm in width and 250 mm in length were fabricated [36,37]. Tests were used to confirm analyses performed on composite repair specimens utilizing two distinct structures, one with seven layers and the other with nine layers. Hexforce G0904 material was used for repairs, whereas M21/AS4C material was used to produce composite specimens [38]. In Table 1, the mechanical characteristics of the main structure and the repair material properties are listed. Table 2 presents the mechanical properties of HYSOL EA 9396 adhesive used in wet lay-up repair on a prepreg parent structure. Table 3 provides the mechanical properties of the core used in the sandwich structure. The core material was only used in areas of the wing structure where there were no beams or ribs in contact with the skin.

#### 2.1.1. Preparation of Test Specimens for Validation

The fabrication and repair procedures for the composite specimens adhered strictly to standardized protocols used in the manufacturing of civil and military aircraft components, as well as their on-site repairs [4]. The composite panels were produced using M21/AS4C carbon/epoxy fabric prepregs, supplied by HEXCEL, a prominent material manufacturer based in Connecticut, USA [39]. This meticulous approach ensures that the specimens are representative of real-world applications, aligning with industry practices and enhancing the validity of the study’s findings. The following is a summary of the procedural sequence:

1. Two panels made of seven layers of carbon fiber oriented as [+45/0/45/0/45/0/+45]_s_ and nine layers oriented as [+45/0/45/0/45/0/45/0/+45]_s_ were produced using M21/AS4C carbon/epoxy fabric prepregs.
2. The two panels were precisely machined using high-rpm CNC machines in accordance with ASTM D3039 standards.
3. After cutting, the samples were subjected to tape inspection and manual ultrasonic pulse-echo (MUPE) inspection to verify the integrity of the panels.
4. For the repair process, material removal was conducted to the depth corresponding to the original carbon layers. The process involved sanding the area with 240-grit or finer sandpaper to prepare a 12 mm overlap region for the repair layers.
5. Following the material removal, the surfaces were meticulously cleaned using a cheesecloth soaked in appropriate solvents.
6. The repair was performed using Hexforce G0904 D 1070 TCT (HEXCEL, Dagneux, France) fabric, a low-temperature curing prepreg system, in conjunction with Hysol EA 9396 (LOCTITE, HENKEL, Düsseldorf, Germany) resin. The repair was cured at a temperature of 66 ± 2 °C for 1 h.
7. After the repair was completed, the panels underwent further non-destructive testing using automatic ultrasonic through-transmission (AUTT) and MUPE inspections. The AUTT inspection revealed a maximum attenuation of 6–7 decibels, which indicates minimal loss of signal strength, suggesting a well-executed repair with no significant internal defects.
8. Upon successful completion of the repair and inspection processes, the samples were marked appropriately and prepared for subsequent mechanical testing.

**Figure 1:**
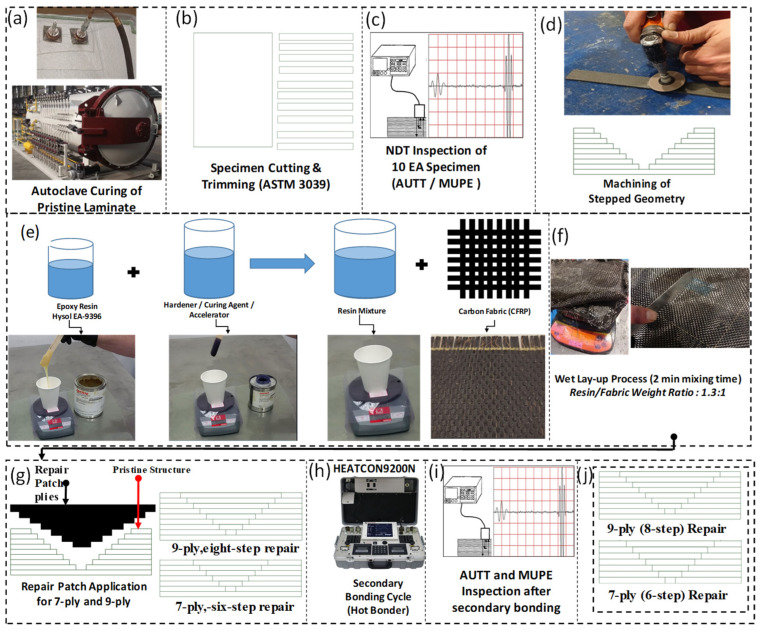
Detailed experimental workflow for the fabrication and stepped-lap repair of CFRP composite panels: (**a**) Autoclave curing of the pristine parent laminate; (**b**) specimen extraction and trimming according to ASTM D3039 standards; (**c**) baseline non-destructive testing (NDT) of the specimens using AUTT and MUPE to ensure structural integrity; (**d**) precision machining of the stepped-lap geometry to create the stepped-lap repair; (**e**) preparation of the adhesive system (Hysol EA-9396 (LOCTITE, HENKEL, Düsseldorf, Germany)) with specific mixing ratios; (**f**) wet lay-up process involving the manual impregnation of carbon fabrics (1.3:1 ratio); (**g**) schematic stacking sequence of the repair plies onto the parent structure; (**h**) secondary bonding cycle controlled by a Heatcon 9200N Hot Bonder; (**i**) post-repair NDT validation to detect interface defects; and (**j**) final architectural schematics of Configuration 2 (9-ply/8-step) and Configuration 1 (7-ply/6-step) specimens.

The comprehensive workflow for specimen fabrication and repair is systematically illustrated in Figure 1. The process began with the fabrication of pristine carbon fiber-reinforced polymer (CFRP) laminates via an autoclave curing cycle to ensure optimal consolidation (Figure 1a). Subsequently, the cured panels were precision-cut into coupon-sized specimens in strict adherence to ASTM D3039 dimensional standards (Figure 1b).

Prior to machining, a baseline quality inspection was conducted using Air-Coupled Ultrasonic Testing (AUTT) and Mu-Pulse Echo (MUPE) to verify structural homogeneity and ensure the absence of initial manufacturing defects (Figure 1c). To simulate the repair scenario, stepped-lap geometries were machined onto the pristine specimens, creating the specific scarf angles required for the joint architecture (Figure 1d).

Concurrently, the adhesive system was prepared by mixing Hysol EA-9396 epoxy resin and hardener for 2 min to ensure stoichiometric homogeneity (Figure 1e). The repair patches were fabricated via a wet lay-up process, where carbon fabric plies were impregnated with the resin mixture at a controlled resin-to-fabric weight ratio of 1.3:1 (Figure 1f). These plies were then stacked onto the scarf region following the ply-drop sequence detailed in Figure 1g.

The secondary bonding cycle was executed using a Heatcon 9200N Hot Bonder (HEATCON Composite Systems, Seattle, WA, USA), providing a controlled thermal environment using a heat blanket under continuous vacuum consolidation to ensure proper compaction (Figure 1h). Post-cure integrity was validated through a secondary NDT inspection to detect any interfacial voids or delaminations (Figure 1i). The final specimen configurations are classified as Configuration 2 (9-ply, 8-step) and Configuration 1 (7-ply, 6-step) as depicted in Figure 1j.

#### 2.1.2. Numerical Modeling of Experimental Samples for Validation

**Figure 2:**
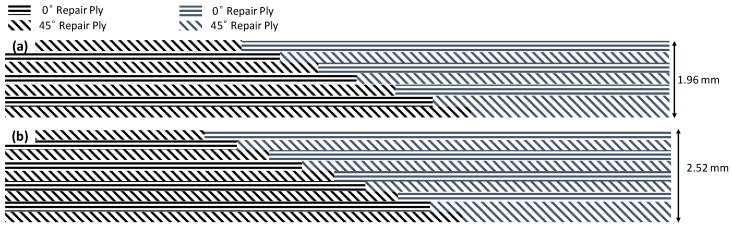
(**a**) 7-layered test specimen and six-step repair (7 ply), Configuration 1 [+45/0/45/0/45/0/+45]; (**b**) 9-ply test specimen and eight-step repair (9 ply), Configuration 2 [+45/0/45/0/45/0/45/0/+45].

The composite repair model shown in Figure 2 was modeled on the specimen along its 12.5 mm width using a shell. The shell was divided using the partition face to assign materials according to the number and orientation of composite layers at the relevant location. Structural analyses were performed using the shell elements specified in Figure 2 for the repair modeling. Instead of using the commonly employed COH3D8 or COH3D20 elements for repair modeling in ABAQUS, this study presents explicit method analyses performed with S4R shell elements. For the shell elements, it was assumed that the thickness of the adhesive remained consistent at each step and there were no undesirable features such as porosity between the parent structure and the repair layers. The materials were systematically introduced into the system from bottom layer to top layer of the sample, adhering to their respective orientations, and the stiffness assignments were applied to the structure according to the designated regions.

The repair patch was constructed using Hexforce G0904 D 1070 TCT carbon fabric combined with Hysol EA 9396 resin via a wet lay-up process. It is important to note that all repair plies (0° and 45°) consist of identical material and thickness; the variation is solely in the fiber orientation to optimize the load-bearing capacity.

### 2.2. Full-Scale Wing Model

**Figure 3:**
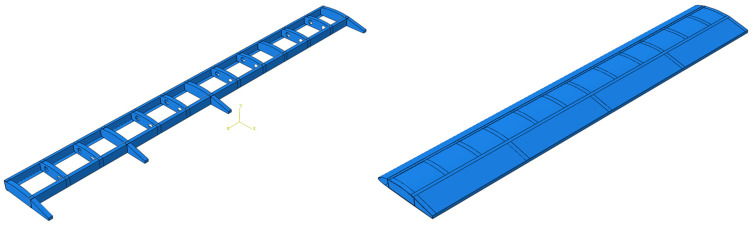
Full-scale wing model that consists of two C spars, eight anti-sloshing ribs, five structural ribs, and four trailing edge ribs.

The full-scale composite wing model shown in Figure 3 is designed according to the properties of M21 AS/4C material. The wing consists of two C spars, eight anti-sloshing ribs, five structural ribs, and four trailing edge ribs. The spanwise length of the composite wing structure is 8000 mm, the chordwise length is 1680 mm, and the height is 365 mm.

The composite wing structure was analyzed with cohesive surface contacts without the use of bolted joints. The structural analysis of the wing structure was performed using ABAQUS/Explicit module. In the structural analyses, the dominant behavior in the curved regions is the bending deformation, resulting from the wing structure’s curved shape and applied loads. However, in this scenario, the elements exhibit unrealistic shear stresses instead of the expected bending stresses [33]. Fully integrated first-order continuum elements exhibit this behavior. As a result, the bending energy of the element is converted into shear deformation, causing the element to behave excessively stiff and leading to shear locking [33,34]. To prevent shear locking, first-order reduced-integration elements, specifically S4R elements, were used in the models. These elements are designed to prevent undesirable deformations and accurately model finite membrane deformations [40].

The loads on the wing model include aerodynamic forces, gravitational forces, ammunition weight, and fuel weight. Aerodynamic forces on the upper and lower surfaces were transferred from ANSYS Fluent 2023 R1 software to ABAQUS for further analysis. The ammunition weight on the wing was determined by selecting the Mini Smart Munition (MAM-L), represented as three separate 22 kg masses placed at distances of 700 mm, 1400 mm, and 2000 mm from the beginning of the spar. The fuel weight within the wing structure was calculated using a fuel density of 810 kg/m^3^. Assuming the first nine compartments of the wing are fully filled with fuel, a pressure of 0.004 MPa was applied to the lower shell structure where the fuel contacts. The total fuel mass in these compartments was calculated to be 637.8 kg, while the last three compartments were left empty. To maintain consistent loading on the wing model, the fuel compartments were divided into four independent sections, each supported by anti-flutter ribs spaced at least 600 mm apart. This arrangement was designed to enhance wing stability and prevent flutter. The structural analysis of the full-scale wing model was initially conducted without any repairs to identify the most critical region on the wing. This analysis aimed to evaluate the structural durability and to identify the potential weak points in the wing structure.

**Figure 4:**
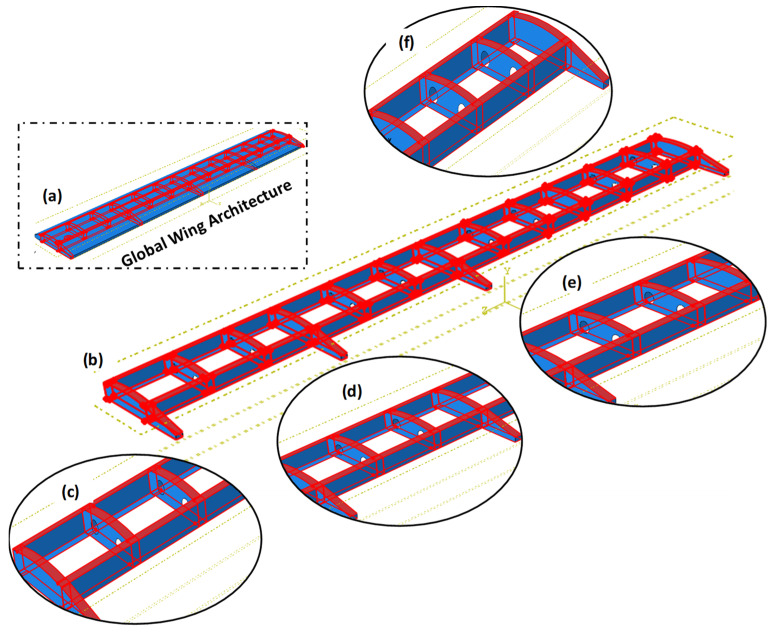
(**a**) Global wing architecture; (**b**) cohesive surface contact areas on full scale wing; (**c**) zone 1 (root/fuselage interface); (**d**) zone 2 (inboard section); (**e**) zone 3 (mid-span section); (**f**) zone 4 (outboard/tip section).

Figure 4 illustrates the advanced bolt-free integrated assembly architecture of the full-scale wing model and explicitly defines the four critical functional zones (Zone 1–4) established for structural integrity assessment. The schematic provides a detailed visualization of: (a) the global wing architecture featuring seamless cohesive integration; (b) the high-fidelity cohesive surface contact areas where the primary load transfer between spars, ribs, and the wing skin occurs; (c) Zone 1, representing the high-stiffness wing-to-fuselage root interface; (d) Zone 2, the inboard segment located between the first set of sloshing and structural ribs; (e) Zone 3, the central mid-span section defined by subsequent rib sets; and (f) Zone 4, the outboard wing tip section situated beyond the terminal sloshing and structural ribs.

The decision to analyze the full-scale wing assembly without mechanical bolted joints represents a deliberate architectural choice, reflecting a paradigm shift toward “all-bonded” integrated composite structures in modern aerospace design. Traditional fasteners are known to introduce parasitic stress concentrations and fiber discontinuities; thus, a purely cohesive interface was utilized to achieve a more uniform “shear lag” distribution. This approach ensures that the repair does not introduce secondary stiffness mismatches—often termed “parasitic stiffness”—which typically occur at bolt−hole interfaces and can lead to localized numerical singularities irrelevant to the repair’s primary performance. By employing a cohesive surface methodology, a continuous and clean load path is maintained through the wing skin, allowing for a high-fidelity evaluation of secondary bending and peeling stresses without interference from secondary mechanical fastener effects.

The numerical framework for the cohesive interface is based on validated strategies where the Benzeggagh−Kenane (B-K) criterion was selected over the Power Law due to its superior accuracy in predicting mixed-mode failure characteristics in aerospace-grade CFRP joints. The B-K exponent was established at 1.45, aligned with the established literature for brittle adhesive systems. To accurately reflect the fracture energy dissipation observed in experimental stepped repairs, a linear energy-based evolution law was implemented, supported by the energy-dissipation models found in recent studies.

The wing skin and repair patch were discretized using S4R elements, which provide significant computational efficiency for full-scale structures without sacrificing the capacity to capture out-of-plane bending and “kicking” effects. To suppress zero-energy deformation modes inherent in reduced-integration shells, an enhanced Hourglass Control was utilized. The model successfully captures non-linear geometric effects, such as secondary bending, while maintaining computational robustness.

Consistent stiffness distribution across the assembly was ensured through a hierarchical contact selection, designating the spars and ribs as the first surface and the skin as the second surface. Furthermore, a viscosity coefficient of 0.001 was implemented for damage stabilization. This provides a robust numerical bridge between the localized initiation, predicted by the Maximum Nominal Stress (MAXS) criterion, and the ultimate unstable cohesive separation observed during experimental verification.

### 2.3. Determination of Aerodynamic Forces

Computational fluid dynamics (CFD) simulations were employed to determine the aerodynamic loads on the composite wing structure at various angles of attack. A freestream velocity of 0.5 Mach was selected, considering the maximum velocity of the aircraft. The airfoil used has a maximum thickness equal to 14.4% of the chord length, located at 38.8% of the chord length from the leading edge. Additionally, the maximum camber is also positioned at 38.8% of the chord length. The governing equations for the simulations are the Navier–Stokes and continuity equations, which are presented as Equation (1) and Equation (2), respectively [41,42]. In these equations, **v** denotes the flow velocity, **ρ** represents the mass density of the fluid, **τ** symbolizes the fluid stress, and **t** denotes the time:

(1) $$\rho \frac{\partial v}{\partial t}+\rho (v)\cdot \nabla v-\nabla \cdot \tau =0$$

(2) ∇⋅v=0

**Figure 5:**
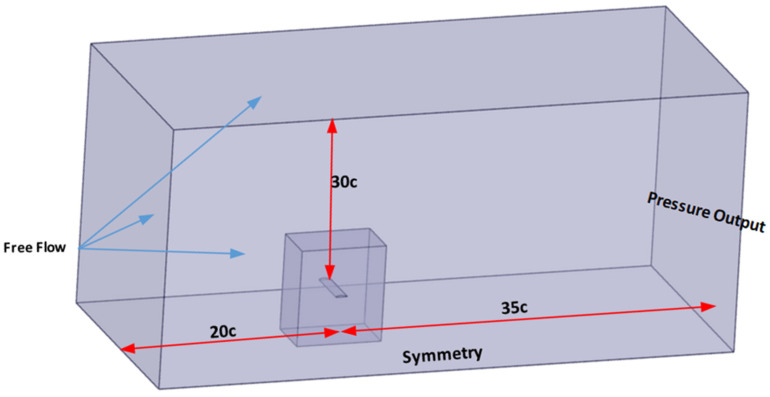
The geometric model of the CFD model.

To focus on the primary factors influencing aerodynamic performance, gravitational effects were neglected. Although the fluid domain was modeled as a compressible ideal gas to capture flow variations at Mach 0.5, the wing surface was treated as an adiabatic wall, assuming no heat transfer across the fluid–structure interface. The continuity equation, expressed in Equation (2), ensures the conservation of mass within the simulated fluid domain, which is crucial for accurately capturing the flow behavior around the composite wing structure. This approach allows for the precise assessment of the aerodynamic characteristics under varying conditions, providing valuable insights into the performance of the composite material under different loading scenarios. The geometric model of the CFD simulations is provided in Figure 5.

The control volume shown in Figure 5 is designed by considering two nested volumes. The smaller inner volume is utilized to create fine elements around the wing for fuselage sizing. The larger control volume is designed in the shape of a rectangular prism. The dimensions of this rectangular prism have been determined based on the chord length of the wing, in accordance with the relevant literature [43,44]. In the conducted CFD analyses using ANSYS Fluent software, the computed thickness of the first fluid layer was found to be 2.9 × 10^−5^ m to ensure an accurate boundary layer resolution and aerodynamic load transfer.

The numerical analysis was conducted under operating conditions corresponding to an altitude of 35,000 ft based on the International Standard Atmosphere (ISA). The freestream flow was defined with a Mach number of 0.5 ($V_{\infty }$ = 148.3 m/s). To capture compressibility effects, the fluid was modeled as an ideal gas with a freestream density of 0.380 kg/m^3^ and a static pressure of 23,842 Pa. The dynamic viscosity was governed by Sutherland’s law, with a reference freestream value of 1.43 × 10^−5^ kg/(m·s) corresponding to an ambient temperature of 218.8 K.

### 2.4. CFRP Failure Criteria

The failure criteria that have been verified by the theories and experiments must be compared with the stress condition of the material to determine the structural integrity and efficacy of materials used in the repaired structures. The load-bearing capacity and the damage initiation of composite laminates may be precisely predicted by the Hashin failure criterion and Yeh delamination [45,46,47]. Inter-laminar failure in stepped-lap repair is an unlikely scenario under uniaxial tension [17]. Therefore, in the CFRP laminates under loading, fiber failure, matrix failure, and delamination damage are predicted at the integration points using the Hashin failure criterion using Equations (3)–(8) [4].

Hashin failure criterion for tensile fiber fracture ($\sigma_{11}$ > 0) is described in Equation (3):

(3) $${\left(\frac{\sigma_{11}}{X_{T}}\right)}^{2}+{\left(\frac{\tau_{12}}{S_{12}}\right)}^{2}+{\left(\frac{\tau_{13}}{S_{13}}\right)}^{2}=1$$

Hashin failure criterion for tensile fiber fracture ($\sigma_{11}$ < 0) is given in Equation (4):

(4) $${\left(\frac{\sigma_{11}}{X_{C}}\right)}^{2}=1$$

Hashin failure criterion for the tensile fiber fracture ($\sigma_{22}+\sigma_{33}$ > 0) is defined in Equation (5):

(5) $${\left(\frac{\sigma_{22}+\sigma_{33}}{Y_{T}}\right)}^{2}+\frac{\left(\tau_{23}^{2}-\sigma_{22}\sigma_{33}\right)}{S_{12}^{2}}+{\left(\frac{\tau_{12}}{S_{12}}\right)}^{2}+{\left(\frac{\tau_{13}}{S_{13}}\right)}^{2}=1$$

Hashin failure criterion for the tensile fiber fracture ($\sigma_{22}+\sigma_{33}$ < 0) is provided in Equation (6):

(6) $$\frac{\left(\sigma_{22}+\sigma_{33}\right)}{Y_{C}}\left[{\left(\frac{Y_{C}}{2S_{23}}\right)}^{2}-1\right]+{\left[\frac{\left(\sigma_{22}+\sigma_{33}\right)}{2S_{23}}\right]}^{2}+\frac{\left(\tau_{23}^{2}-\sigma_{22}\sigma_{33}\right)}{S_{23}^{2}}+{\left(\frac{\tau_{12}}{S_{12}}\right)}^{2}+{\left(\frac{\tau_{13}}{S_{13}}\right)}^{2}=1$$

The Yeh delamination failure criteria can be described as given in Equation (7) for the tensile delamination mode ($\sigma_{33}$ > 0), and in Equation (8) for the shear delamination mode ($\sigma_{33}$ > 0):

(7) $${\left(\frac{\sigma_{33}}{Z_{T}}\right)}^{2}+{\left(\frac{\tau_{13}}{S_{13}}\right)}^{2}+{\left(\frac{\tau_{23}}{S_{23}}\right)}^{2}=1$$

(8) $${\left(\frac{\tau_{13}}{S_{13}}\right)}^{2}+{\left(\frac{\tau_{23}}{S_{23}}\right)}^{2}=1$$

In the given equations, $\sigma_{11}$, $\sigma_{22}$, and $\sigma_{33}$ denote the element’s stress values in three directions, and $\tau_{12}$, $\tau_{23}$, and $\tau_{13}$ are the shear stresses in the fiber direction, transverse direction, and thickness direction, respectively. The three components of CFRP’s shear strength are represented by the parameters $S_{12}$, $S_{13}$, and $S_{23}$, respectively. The tensile strength of the composite material is represented by the numbers $X_{T}$, $Y_{T}$, and $Z_{T}$ in the fiber direction, transverse direction, and thickness direction, respectively. The compressive strength of CFRP is represented by the parameters $X_{C}$, $Y_{C}$, and $Z_{C}$ in the fiber direction, transverse direction, and thickness direction, respectively [4,26].

### 2.5. Repair Scenarios on the Full-Scale Wing Model

**Figure 6:**
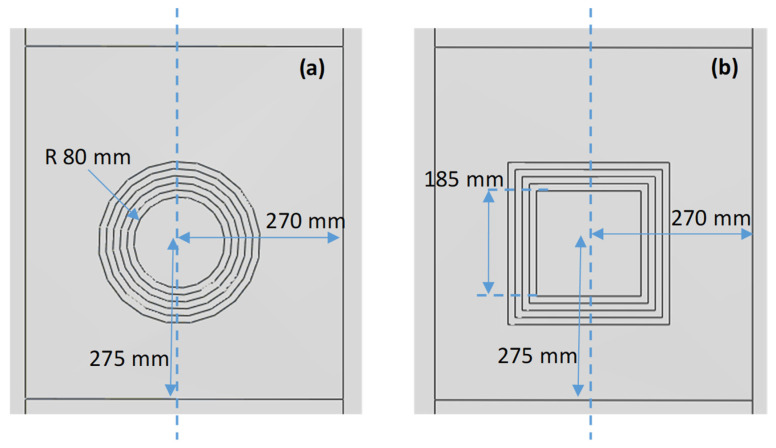
The geometric dimensions of repair models. (**a**) The circular repair model. (**b**) The square repair model.

Multiple repair scenarios were implemented on the full-scale wing model, primarily categorized into circular and square repair designs. The geometric dimensions related to the circular and square repairs are provided in Figure 6. The distance from the center of the designed repairs to the spar is modeled as 270 mm, while the distance to the rib is modeled as 275 mm. The dimensions of each repair layer have been extended by 12.5 mm from the edges according to the respective geometric shape [48].

Table 4 presents a comparison of scenarios for the circular and square repaired structures of single-region repairs. The scenarios include the damaged and repaired forms of the structures in order to elucidate the effect of repairs. The main differences between the repair scenarios are mainly due to the orientation and number of layers. It should be noted that there is no repair patch in Scenario 1.

**Figure 7:**
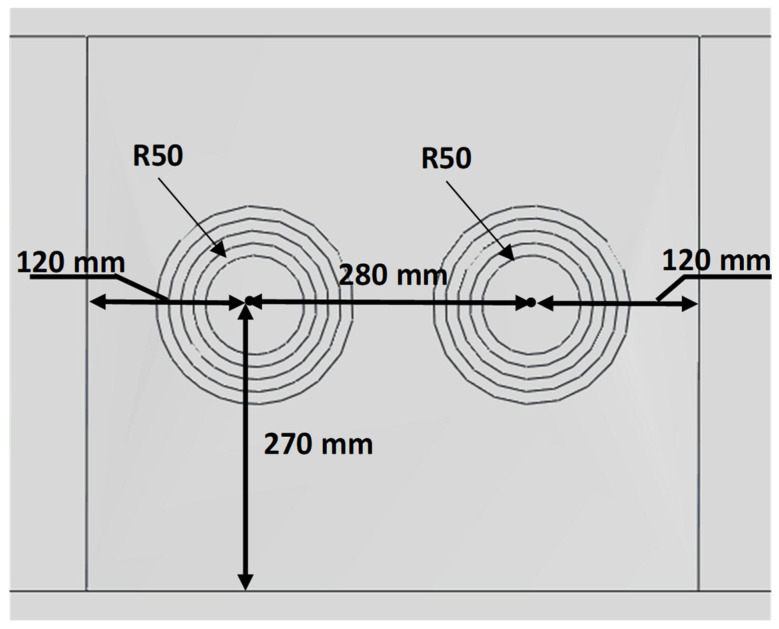
The geometric dimensions of the double repair scenarios.

In addition to the studies focusing on single-region repairs, the cases involving two closely located damaged areas were also examined. For these scenarios, the circular repair design was identified as the optimal approach. Figure 7 illustrates the double circular repair design implemented in the most critical region of the composite wing model analysis. The damaged and repaired scenarios, and related geometrical dimensions of the double repairs, are presented in Table 5. Similar to the single-region repair cases, double repair cases include various orientations and numbers of layers.

## 3. Results and Discussion

In this section, the key findings of numerical analyses and experiments are presented. The results are discussed sequentially, starting with the experimental validation of the shell method, followed by the CFD results, and then the determination of the critical location on the full-scale wing model. Subsequently, the results of repair scenarios on the full-scale wing are explored, beginning with single repairs and followed by double repairs.

### 3.1. Experimental Validation of Shell Method

**Figure 8:**
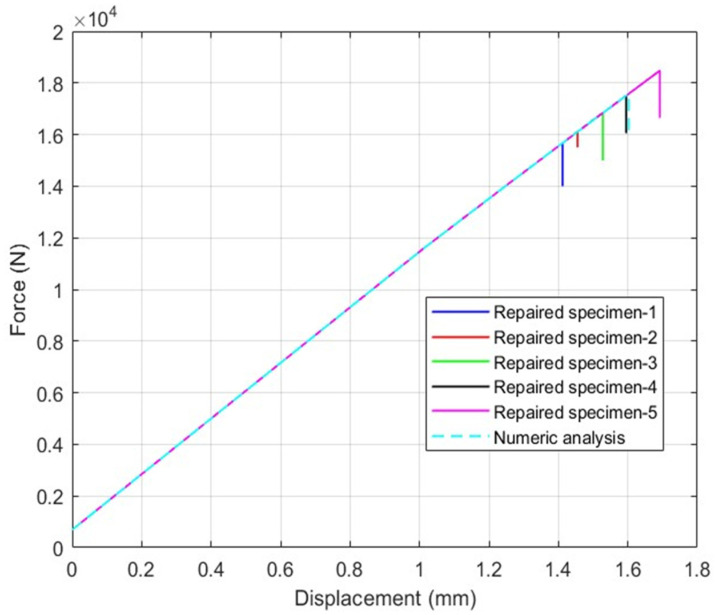
7-layered test specimen results and numeric analysis results using shell method and six-step repair (7 ply), Configuration 1 ([+45/0/45/0/45/0/+45]).

The experimental results of test specimens and the results of the numerical models are summarized and compared in Table 6. The deviation between the test results and the numerical model results yielded a stress difference of less than 10% across all cases. Test findings given in Table 6 indicate that cohesive and intralaminar failure modes exist, as shown in Figure 8. The observed failure modes have been shown to be in agreement with the numerical findings.

**Figure 9:**
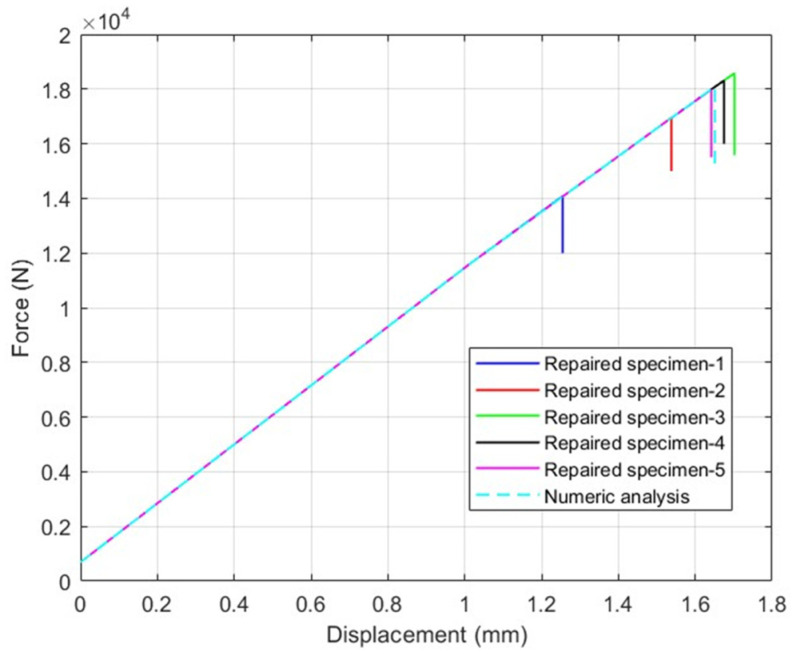
9-layered test specimen results and numeric analysis results using shell method and eight-step repair (9 ply), Configuration 2 ([+45/0/45/0/45/0/45/0/+45]).

In Figure 8 and Figure 9, the test results obtained for the repaired specimens and the analysis results for the repair models are presented for 7-layered and 9-layered test specimens, respectively. The test results for the average tensile stress and average strain values show similarities with the analysis results. However, considering that the loads experienced by the layers may vary depending on their orientation, evaluations in the composite wing model were performed based on the strain values. In Figure 7, it is observed that failure points of the tests vary within the displacements of 1.25 mm and 1.7 mm. The reason for this difference can be explained by the composite fabrication quality. Similar differences in the test results are also observed in Figure 8. The findings of the numerical models are obtained within the range of test results. This result shows that the numerical models adopting the shell method can realistically model the experimental tests of repaired specimens.

**Figure 10:**
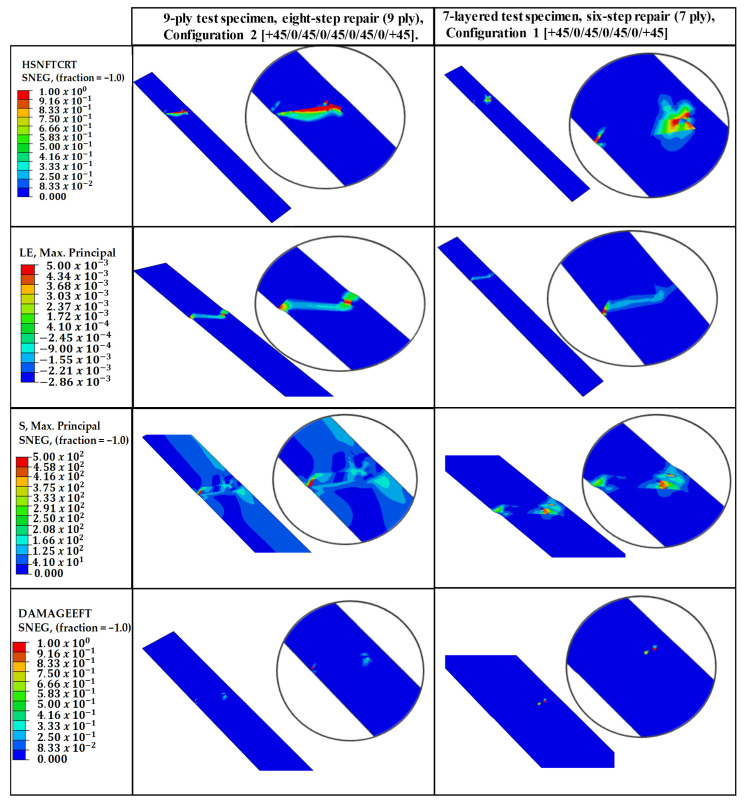
Comparative numerical field outputs for the 9-ply (Configuration 2) and 7-ply (Configuration 1) stepped-lap repair specimens under tensile loading. The contours illustrate the progression of failure mechanisms: (Row 1) HSNFTCRT: Hashin fiber tensile damage initiation criterion, identifying critical failure onset at the step corners due to stress singularities; (Row 2) SNEG: Strain energy density distribution, highlighting the energy localization zones induced by secondary bending; (Row 3) $S_{11}$: Longitudinal stress distribution demonstrating the load transfer efficiency and stress concentrations at the repair overlap; and (Row 4) DAMAGEFT: Evolution of fiber tensile damage, visualizing the propagation of structural degradation following the initial failure.

The fidelity of the finite element model in capturing the localized failure phenomena is substantiated by the field output distributions presented in Figure 10. The analysis compares the structural response of the 9-ply and 7-ply configurations, revealing a distinct correlation between geometric discontinuities and damage initiation.

The Hashin Fiber Tension Initiation (HSNFTCRT) results accurately predict that failure initiates localized fiber breakage. This numerical onset is physically validated by Scanning Electron Microscopy (SEM) images, which provide evidence of initial fiber fractures at the predicted stress concentration zones.

The structural transition from initiation to ultimate failure is explained by the localized nature of the fiber damage. Minimal values observed in the DAMAGEFT (Fiber Tension Damage) field outputs indicate that the failure is not a result of a gradual or slow progression of fiber degradation. Instead, the localized fiber initiation (HSNFTCRT) acts as a structural “trigger”. Once the failure threshold is reached (1.0), an immediate and unstable load redistribution to the adhesive interface occurs. 

**Figure 11:**
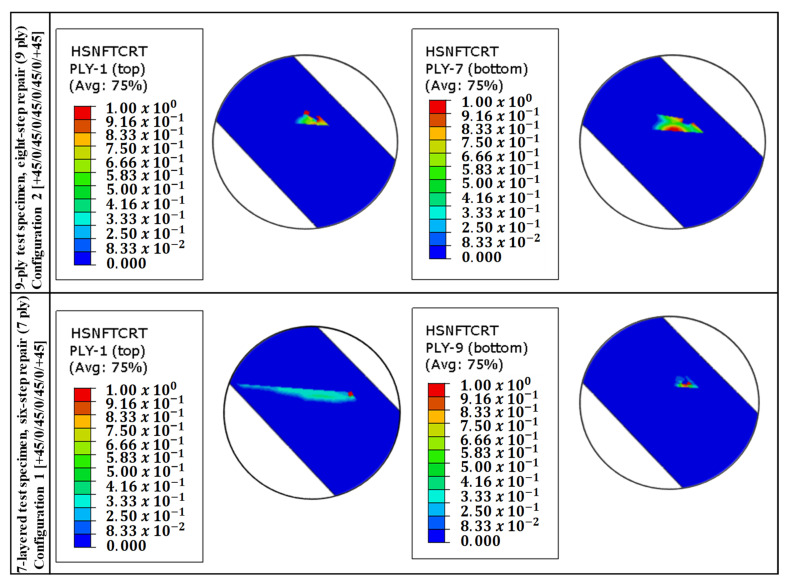
Through-thickness variation in fiber tensile damage initiation (HSNFTCRT) indices for Configuration 2 (9-ply) and Configuration 1 (7-ply).

**Figure 12:**
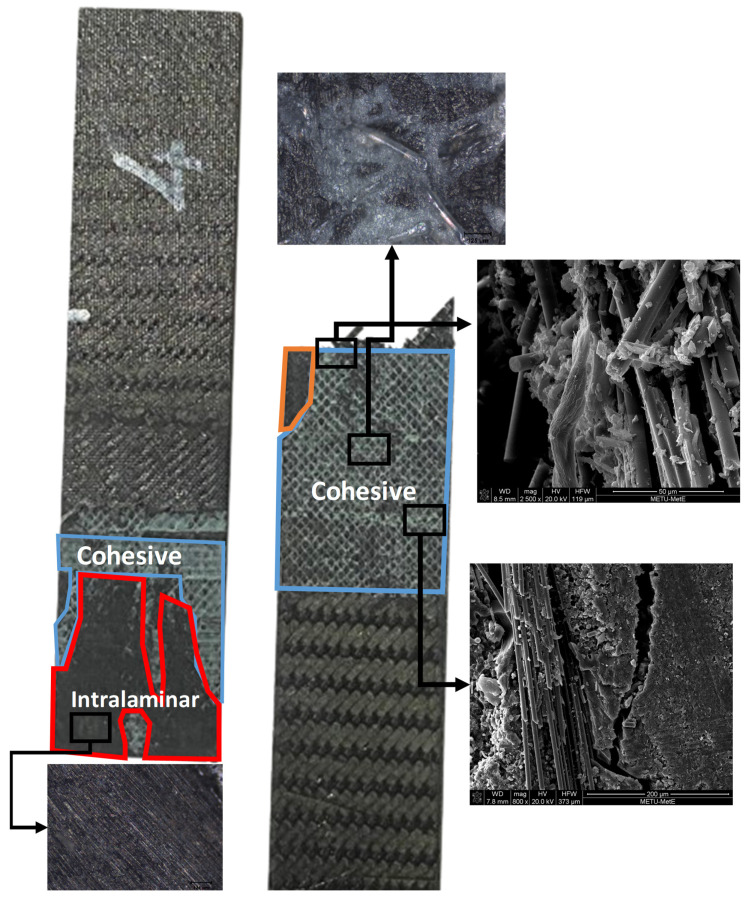
High-powered optical microscope and SEM images of fracture morphology of CFRP six-step repair (7-layered specimen), Configuration 1 ([+45/0/45/0/45/0/+45]).

**Figure 13:**
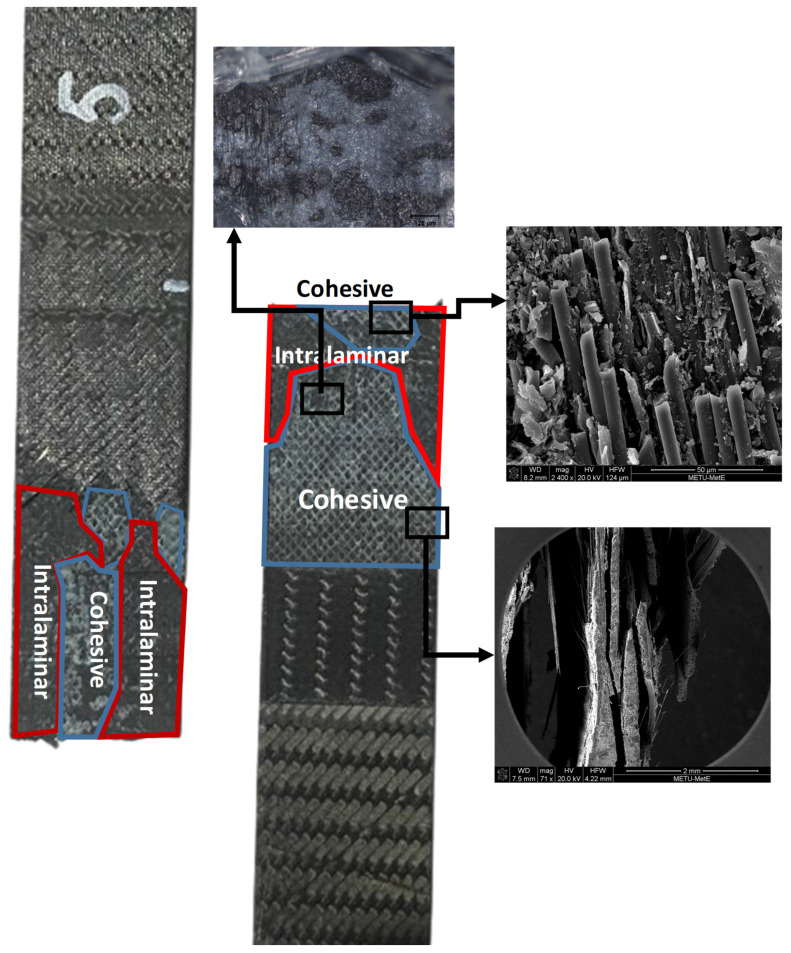
High-powered optical microscope and SEM images of fracture morphology of CFRP eight-step repair (9-layered specimen), Configuration 2 ([+45/0/45/0/45/0/45/0/+45]).

While the numerical results for Hashin Fiber Tension Initiation (HSNFTCRT) exhibit high concentrations at the top edge—consistent with the anticipated secondary bending effects—the failure process is fundamentally triggered by the fiber phase, as indicated in Figure 11. This mechanism explains the experimental observation in Figure 12 and Figure 13, where a high-index fiber initiation area rapidly transitions into a widespread cohesive debonding zone, preventing further fiber damage progression.

The repair architectures investigated in this study, specifically the 7-ply (1.96 mm) and 9-ply (2.52 mm) configurations, exhibit a structural response governed by the neutral axis offset between the parent laminate and the asymmetric repair patch. Although the stepped-lap design theoretically provides a more gradual load transfer than a simple lap, the single-sided application induces a parasitic bending moment (M=P×e) under global tensile loading (P). This eccentricity (e) forces the joint into a state of secondary bending, which is the primary driver for failure initiation.

The field outputs compare the damage state at the Top Surface (Ply-1) versus the Bottom Surface, revealing the structural asymmetry induced by the load path eccentricity. The localized high-index regions (red zones) on the outer plies confirm the significant influence of secondary bending moments, which generate differential tensile stresses across the laminate thickness at the repair overlap.

The presence of secondary bending is numerically confirmed by the through-thickness damage gradient presented in Figure 11. In a pure tensile regime, the stress distribution across the laminate thickness would remain uniform. However, the simulation reveals a distinct asymmetry: high-intensity failure initiation (HSNFTCRT) is observed on the Top Surface (Ply-1), which corresponds to the convex side of the bent specimen where tensile strains are amplified by the curvature moment. Conversely, the Bottom Surface exhibits a different damage topology, consistent with the concave side where the bending-induced compressive vectors partially counteract the global tensile load. This differential failure state between the outer surfaces serves as a quantitative fingerprint of the ‘kicking’ phenomenon caused by the load path eccentricity.

The evaluation of both 7-ply and 9-ply configurations reveals a significant correlation between the top (SPOS) and bottom (SNEG) integration points. For both structures, HSNFTCRT values are notably higher in the top plies compared to the bottom plies. This through-thickness asymmetry confirms that the joint is not subjected to pure tension but undergoes a rotational “kicking” motion characteristic of eccentric stepped-lap joints. The top plies, located furthest from the neutral axis, experience the highest combined axial tensile and bending-induced peeling stresses, thus reaching the failure threshold prematurely.

Although the SPOS layers reach initiation earlier due to peak bending, the SNEG (bottom surface) results are prioritized for the final validation and reporting. This decision is based on the fact that the SNEG integration points reside at the critical interface where load transfer to the parent structure occurs. Numerical validation proves that the localized stress “hot-spots” at the SNEG layer are the truest predictors of the subsequent catastrophic cohesive separation observed in experimental tests. By focusing on the SNEG layer, the model captures the mechanical bridge between intralaminar fiber failure and interfacial debonding. Furthermore, utilizing SNEG outputs provides a more conservative and structurally relevant assessment of the repair’s integrity, ensuring that the predicted load-bearing capacity accounts for the critical interface most susceptible to global structural separation.

The images in Figure 12 reveal the detailed microstructural characteristics of the fracture surfaces, highlighting the effectiveness of the repair technique and the integrity of the fiber−matrix interface. Similarly, the images in Figure 13 demonstrate the fracture patterns and damage mechanisms in the repaired structure, providing insights into the performance and durability of the eight-step repair method.

### 3.2. CFD Simulations

**Figure 14:**
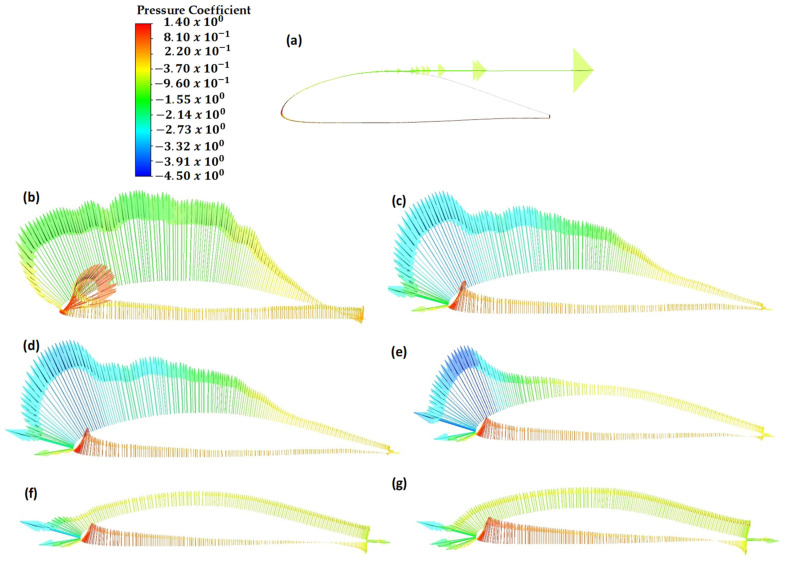
Pressure vectors obtained for various angles of attack. (**a**) 0° angle of attack. (**b**) 5° angle of attack. (**c**) 10° angle of attack. (**d**) 15° angle of attack. (**e**) 20° angle of attack. (**f**) 25° angle of attack. (**g**) 30° angle of attack.

**Figure 15:**
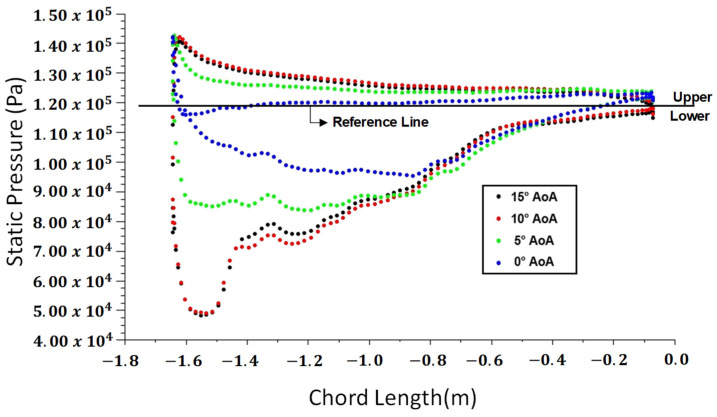
Pressure values along the vector length are shown for various angles of attack: 0°, 5°, 10°, and 15°. The upper part of the reference line represents the static pressure distribution on the lower surface of the airfoil, while the lower part of the reference line represents the static pressure distribution on the upper surface of the airfoil.

**Figure 16:**
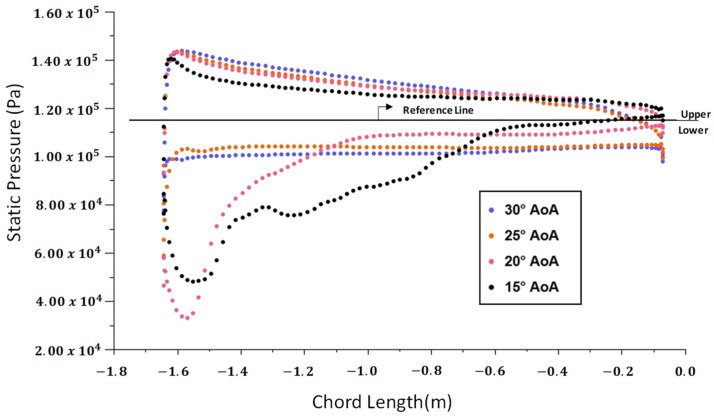
Pressure values along the vector length are shown for various angles of attack: 15°, 20°, 25°, and 30°. The upper part of the reference line represents the static pressure distribution on the lower surface of the airfoil, while the lower part of the reference line represents the static pressure distribution on the upper surface of the airfoil.

Computational fluid dynamics (CFD) analyses provide detailed insights into the behavior of pressures for different angles of attack. This section presents the results of CFD simulations, offering a comprehensive understanding of the aerodynamic characteristics of the modeled full-scale wing model. Considering different angles of attack, the pressure vectors are visually depicted on the airfoil profile in Figure 14. These visual representations help us to understand the variation in the pressure distributions around the airfoil for various angles of attack. The results obtained indicate that pressure levels and distributions on the wing are highly dependent on the angle of attack. In Figure 15, the pressure levels on the upper and lower sides of the wing are compared for the angles of attack ranging from 0 to 15°. It is observed that an increase in the angle of attack results in an increase in the pressure levels. In Figure 16, the angles of attack within 15° to 30° are investigated. When compared with the other angles of attack, the highest-pressure loads on the upper surface are observed at the angles of 25° and 30°.

The results reveal that the lift force generated by the wing begins to decrease when the angle of attack exceeds 15°. A significant drop in the lift force occurs at 25°, resulting in stall conditions. During the potential high-maneuver conditions, the angle of attack could increase up to 30°, where the wing experiences the maximum loading in terms of pressure.

The highest aerodynamic pressure loads observed at a 30° of angle of attack are used in the full-scale wing models in order to model the most critical aerodynamic conditions. The non-uniform pressure distributions on the upper and lower sides are recorded using ANSYS Fluent and transferred to ABAQUS as pressure boundary conditions of the full-scale wing model.

### 3.3. Determination of the Critical Location on the Wing

**Figure 17:**
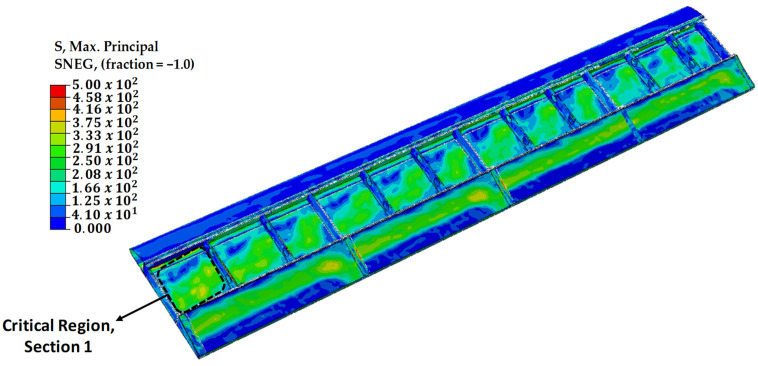
Maximum principal stress (MPa) distribution on the full-scale wing model.

The previously determined aerodynamic pressures and the weights of fuel and ammunition are applied to the full-scale wing model in order to determine the most critical location on the wing in terms of mechanical stresses. The stress distribution on the wing is shown in Figure 17. The wing’s total length is divided into four equal parts, with each part referred to as a section. As expected, the most critical region is determined as Section 1, which is closest to the plane body. In order to ensure the most challenging conditions, all modeling and repair scenarios were conducted in Section 1, the region where the highest mechanical stresses are observed.

The modeled scenarios on the wing model are divided into four parts, as previously summarized in Table 4 and Table 5. These scenarios include the damaged structure analysis, round patch repair analysis, square patch repair analysis, and double round patch repair analysis. In all analyses, the results obtained in the region of Section 1 are presented, including the stress resistance, initiation of shear damage, strain values, and Hashin fiber damage. These results have been used to evaluate the structural integrity and analyze the effects of structural behaviors on the wing.

### 3.4. Results of Repair Scenarios on the Full-Scale Wing Model

In this section, the results of damaged and repaired scenarios are presented and compared to reveal the effects of composite repair strategies.

#### 3.4.1. Single Repairs

Modeled scenarios for single repairs were previously summarized in Table 4. All the repairs are applied to the most critical region (Section 1) on the wing. Consequently, the results presented in this section focus on this critical area. The maximum tensile strain values determined for the scenarios given in Table 4 are presented in Table 7. The maximum tensile strain is observed for Scenario 1, which has damage in three layers with no repair. When the first two layers are repaired as presented in Scenario 2, the maximum tensile strain was significantly reduced, indicating the positive effect of the composite repair process. In Scenario 3, all three damaged layers are repaired and the maximum tensile strain is observed around 2750 με, which is lower than the maximum tensile strain of Scenario 2. This shows that repairing all the damaged layers further improves the structural integrity.

The case with the original condition with no damage or repair was designated as Scenario 5. When the results of Scenario 3 and Scenario 5 are investigated, maximum tensile strains are determined as 2754 με and 2631 με, respectively. It is seen that a similar maximum tensile strain is observed for both cases, showing the similarity between the original (no damage) and repaired cases. However, the original case (Scenario 5) has slightly lower maximum tensile strain compared to the repaired case (Scenario 3).

In Scenario 4, an extra layer is added on top of the three repaired layers. This application further increased the structural strength by reducing the maximum tensile strain to 2628 με. Adding two extra layers in Scenario 6 further decreased the maximum tensile strain to 2560 με. However, adding three extra layers in Scenario 7 resulted in an increase in the maximum tensile strain with a value of 3146 με. In Scenario 8, three additional layers with orientations differing from those in Scenario 7 were applied. Despite these modifications, Scenario 8 exhibited a higher maximum tensile strain of 3626 με, indicating a reduction in structural strength compared to Scenario 7. This outcome underscores the critical influence of layer orientation on the mechanical performance of composite repairs, as improper alignment can lead to increased strain and diminished structural integrity. It was observed that adding up to two extra layers enhanced the structural strength, but the inclusion of a third extra layer resulted in a decline in performance. This suggests that while additional layers can initially improve load distribution and stiffness, excessive layering may introduce stress concentrations or disrupt the balance of the composite structure, ultimately reducing its effectiveness.

In the first eight scenarios, repairs were applied using a circular geometry, while Scenarios 9 and 10 utilized a square geometry for composite repair. The results demonstrated that square-shaped repairs led to increased maximum tensile strains of approximately 4500 με, indicating reduced structural strength. This weakness is attributed to stress concentrations forming at the corners of the square geometry, which compromise the integrity of the repaired structure. Consequently, it is concluded that circular geometric repairs are more effective in enhancing structural strength and should be preferred to minimize stress concentrations and optimize performance.

**Figure 18:**
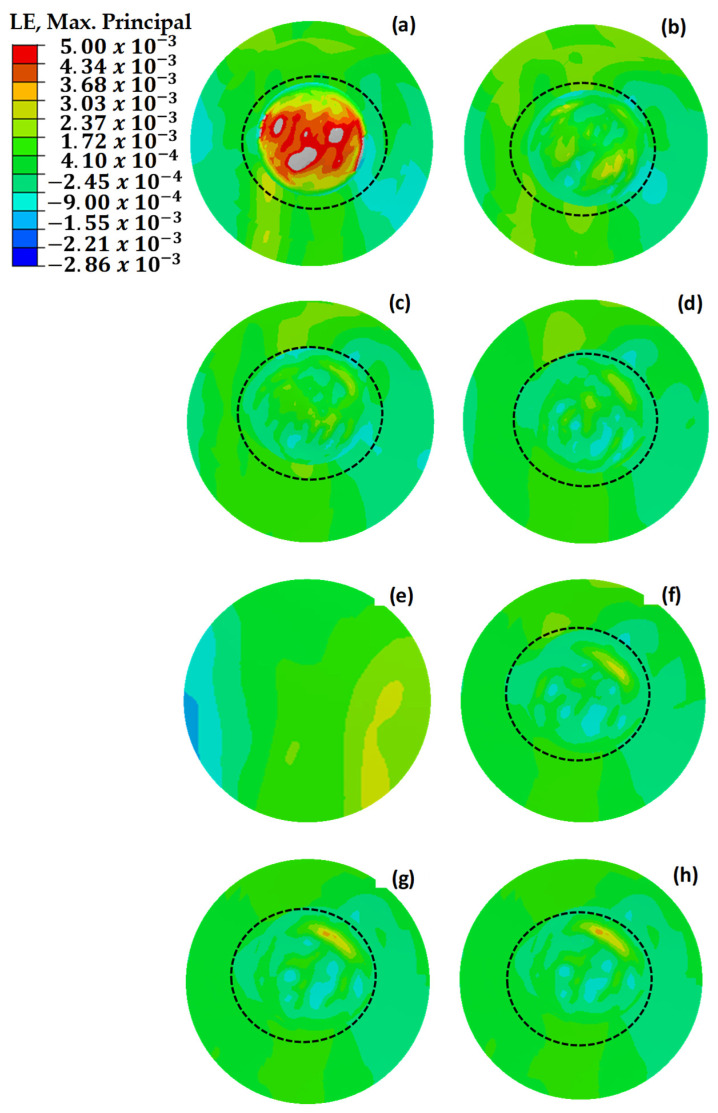
Maximum principal strain (με) values of repaired, pristine, and damaged structures. (**a**) The main structure with 3 missing layers. (**b**) The main structure with 2 missing layers. (**c**) The main structure with 1 missing layer. (**d**) The repair without extra layers. (**e**) The pristine main structure. (**f**) The repaired structure with one extra 45° layer. (**g**) The repaired structure with two extra 45–45° layers. (**h**) The repaired structure with two extra 0–45° layers. The dotted circle delineates the outer limits of the repaired region.

Figure 18 illustrates the maximum principal strain distributions of damaged, pristine, and round-patched cases. The dotted circle indicates the boundary of the repaired region. It is observed that mechanical failure occurred in the structure with three missing layers, as shown in Figure 18a. The repair process conducted without using an additional layer increased the strength by 39% compared to the damaged structure with three missing layers. As the number of missing layers decreased, a reduction was observed in the maximum stresses until reaching the design with an extra layer. With the application of two additional layers, an increase in stress was observed, indicating that the repaired structure could withstand higher loads.

**Figure 19:**
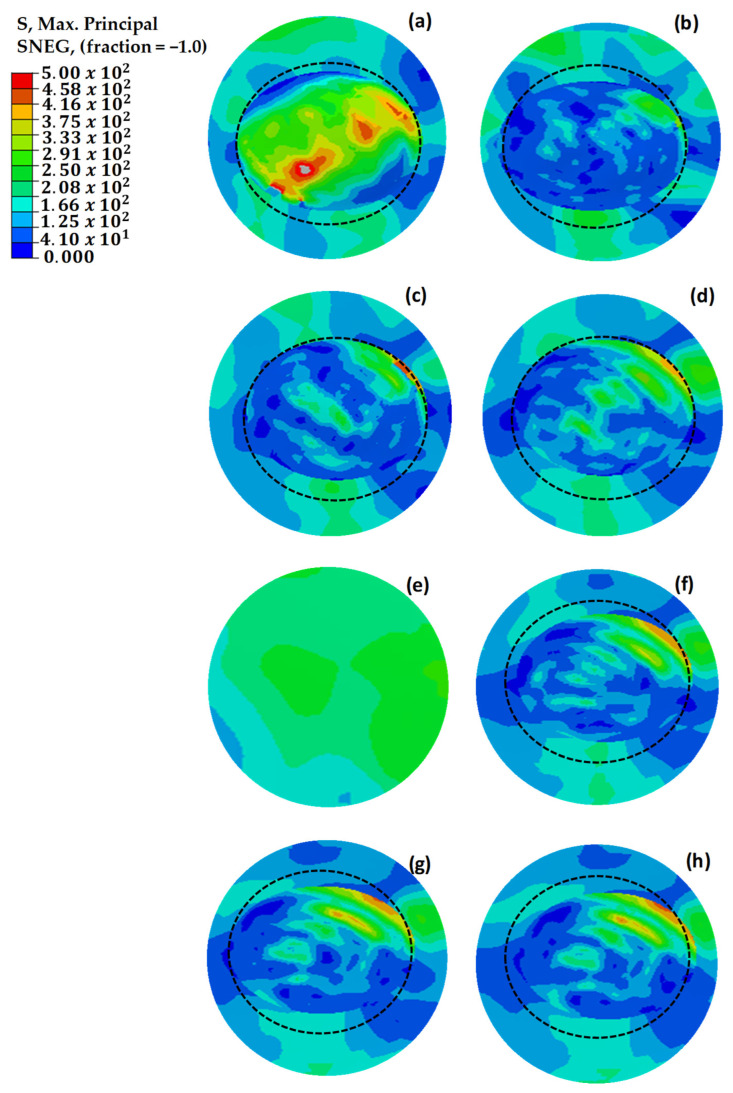
Maximum principal stress (MPa) values of repaired, pristine, and damaged structures. (**a**) The main structure with 3 missing layers. (**b**) The main structure with 2 missing layers. (**c**) The main structure with 1 missing layer. (**d**) The repair without extra layers. (**e**) The pristine main structure. (**f**) The repaired structure with one extra 45° layer. (**g**) The repaired structure with two extra 45–45° layers. (**h**) The repaired structure with two extra 0–45° layers. The dotted circle defines the spatial extent of the repaired region.

The maximum principal stress values depicted in Figure 19 demonstrate uneven distributions in relation to the maximum principal strain values presented in Figure 18. This is due to the variability of stress values based on the orientation of the composite layers, whereas strain values exhibit more consistency across the structure [47,48]. This phenomenon is attributed to the anisotropic nature of the composite material, which exhibits varying strength characteristics depending on the direction of the applied loads. Under load, the composite wing model experienced tensile strains in the bottom skin of the critical region (Section 1), while the top skin underwent compressive stresses. This distinction emphasizes the necessity of considering directional properties in the design and analysis of composite structures to ensure their mechanical integrity under complex loading conditions.

**Figure 20:**
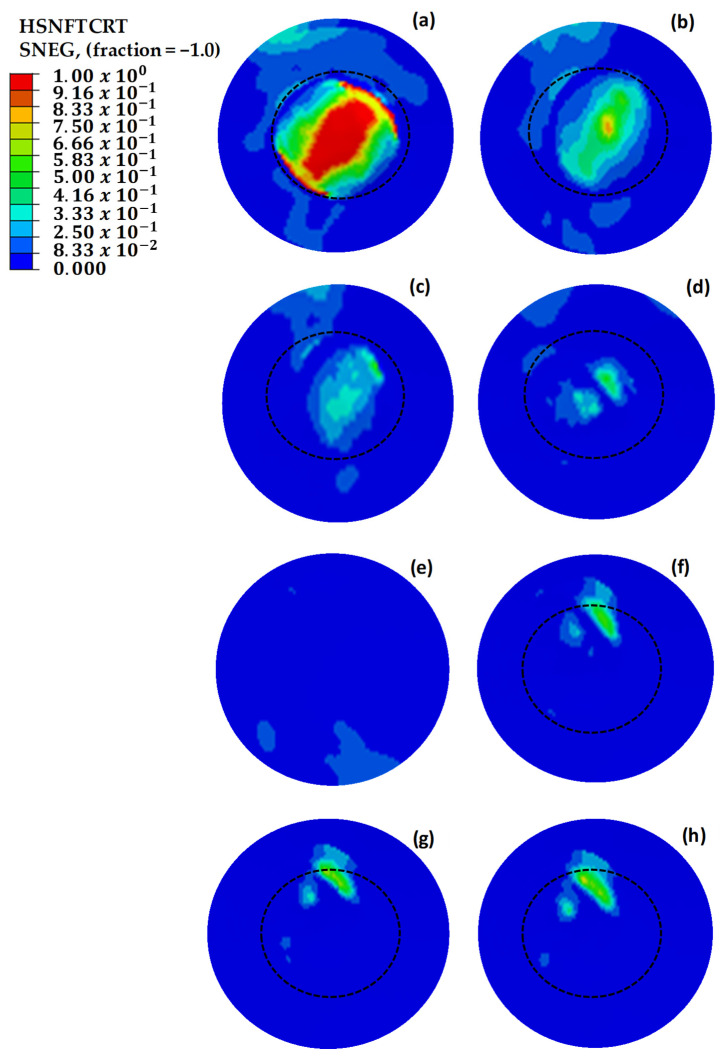
Hashin fiber damage initiation values of repaired, pristine, and damaged structures. (**a**) The main structure with 3 missing layers. (**b**) The main structure with 2 missing layers. (**c**) The main structure with 1 missing layer. (**d**) The repair without extra layers. (**e**) The pristine main structure. (**f**) The repaired structure with one extra 45° layer. (**g**) The repaired structure with two extra 45−45° layers. (**h**) The repaired structure with two extra 0−45° layers. The dotted circle outlines the perimeter of the repaired area.

In the Hashin model analyses performed in the critical region, fiber tensile stress damage was evaluated. Figure 20 illustrates the initial Hashin fiber damage values for damaged, pristine, and circularly repaired designs. The findings from these analyses aligned closely with the results of initial strain, stress, and shear damage assessments, reinforcing the reliability of the Hashin model in predicting damage initiation and progression in composite structures. Consistent with earlier observations, the structure with three missing layers exhibits mechanical failure under load. However, implementing repairs with up to two additional layers effectively enhances the mechanical strength, demonstrating the benefit of controlled layering in restoring structural integrity.

**Figure 21:**
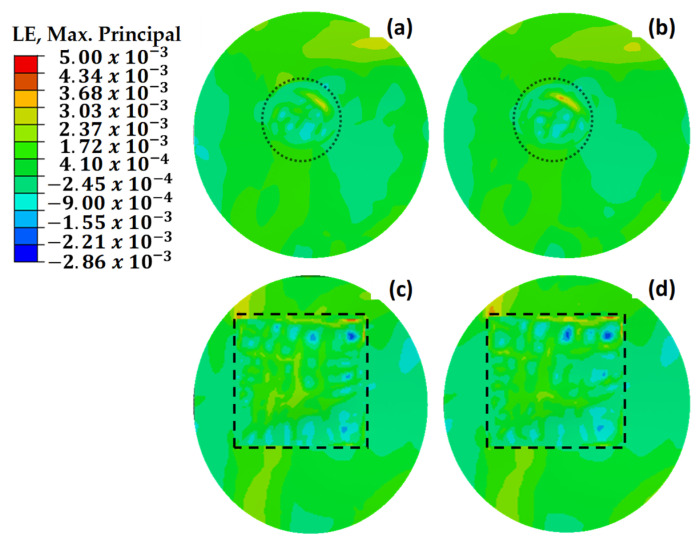
Maximum principal strain (με) values of circular and square repaired structures. (**a**) The circular repaired structure with one extra 45° layer. (**b**) The circular repaired structure with two extra 0–45° layers. (**c**) The square repaired structure with one extra 45° layer. (**d**) The square repaired structure with two extra 0–45° layers. The dotted circle and square outline the boundaries of the repaired area.

Figure 21 compares the strain values of circular and square repaired structures. The circular repair with one additional layer achieved the most effective result, with a strain value of 2560 με. However, adding a second layer to the circular repair increased the observed strain due to the enhanced stiffness. In the square repairs, the strain value with one additional layer was 45% higher than that of the circular repair, highlighting stress concentration at the corners. Adding another layer reduced the strain by 5.5%, as the extra material mitigated the stress discontinuity at the corners. Extending the additional layer by 12.5 mm in the square repair further reduced strain, while a similar extension in the circular repair increased strain due to the absence of corner-induced discontinuities. The added stiffness in the circular repair concentrated more load on the repaired area, leading to higher strain values compared to the surrounding structure.

#### 3.4.2. Double Repairs

In addition to single-region repair studies, scenarios involving two closely located damaged areas were also analyzed. For these cases, the circular repair design was identified as the optimal approach for double repairs due to its superior performance in mitigating stress concentrations and enhancing structural integrity. In Table 8, the maximum tensile strains for the scenarios given in Table 5 are presented. Scenario 11 is the damaged case with three missing layers in both regions. The highest maximum tensile strain is observed in Scenario 11 with a value of 8105 με. In Scenario 12, the first two layers are repaired in two regions, and the maximum tensile strain is reduced to approximately 6000 με. In Scenario 13, all three layers are repaired, and the maximum tensile strain is determined around 5500 με. An extra layer is added in Scenario 14, and the maximum tensile strain is reduced to approximately 5000 με. Scenario 15 refers to the original structure with no damage or repair, and the maximum tensile strain is determined as 2631 με. Scenarios 11, 12, 13, and 14 exhibit significantly higher tensile strain values compared to the original structure (Scenario 15), suggesting that these cases are prone to mechanical failure.

Scenarios 16 and 17, which involve two and three additional layers respectively, show maximum tensile strains of 4673 με and 4539 με. These values indicate that adding up to three layers improves the mechanical strength of the structure in double repair cases, but it remains significantly lower than the strength of the original structure. Furthermore, it is observed that repairs in two closely located regions carry higher risks, and the effect of the repairs and additional layers is more limited compared to single-region repairs.

**Figure 22:**
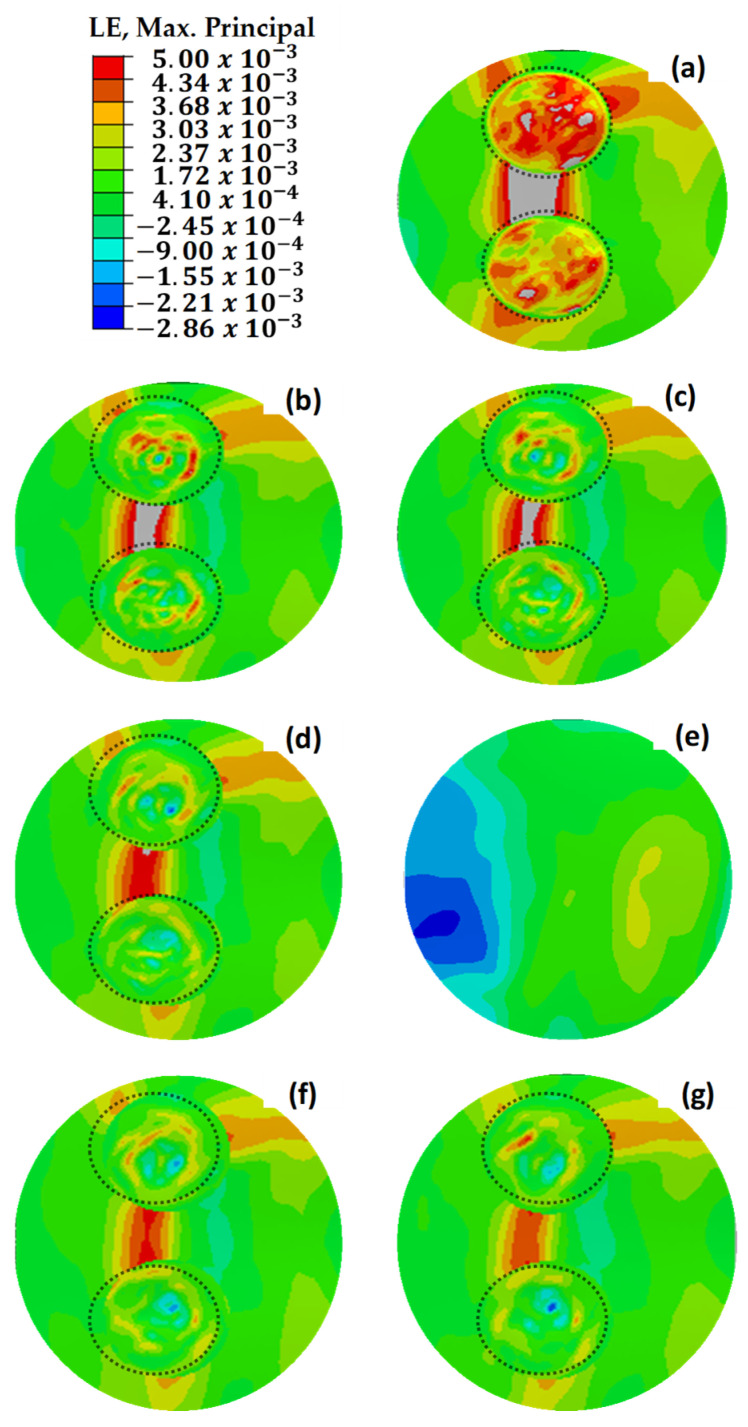
Maximum principal strain (με) values of repaired, pristine, and damaged structures. (**a**) The double main structure with 3 missing layers. (**b**) The double main structure with 2 missing layers. (**c**) The double main structure with 1 missing layer. (**d**) The repair of the double structure without extra layers. (**e**) The pristine main structure. (**f**) The repaired structure with one extra 45° layer. (**g**) The repaired structure with two extra 0–45° layers. The dotted circles indicate the boundaries of the repaired regions.

**Figure 23:**
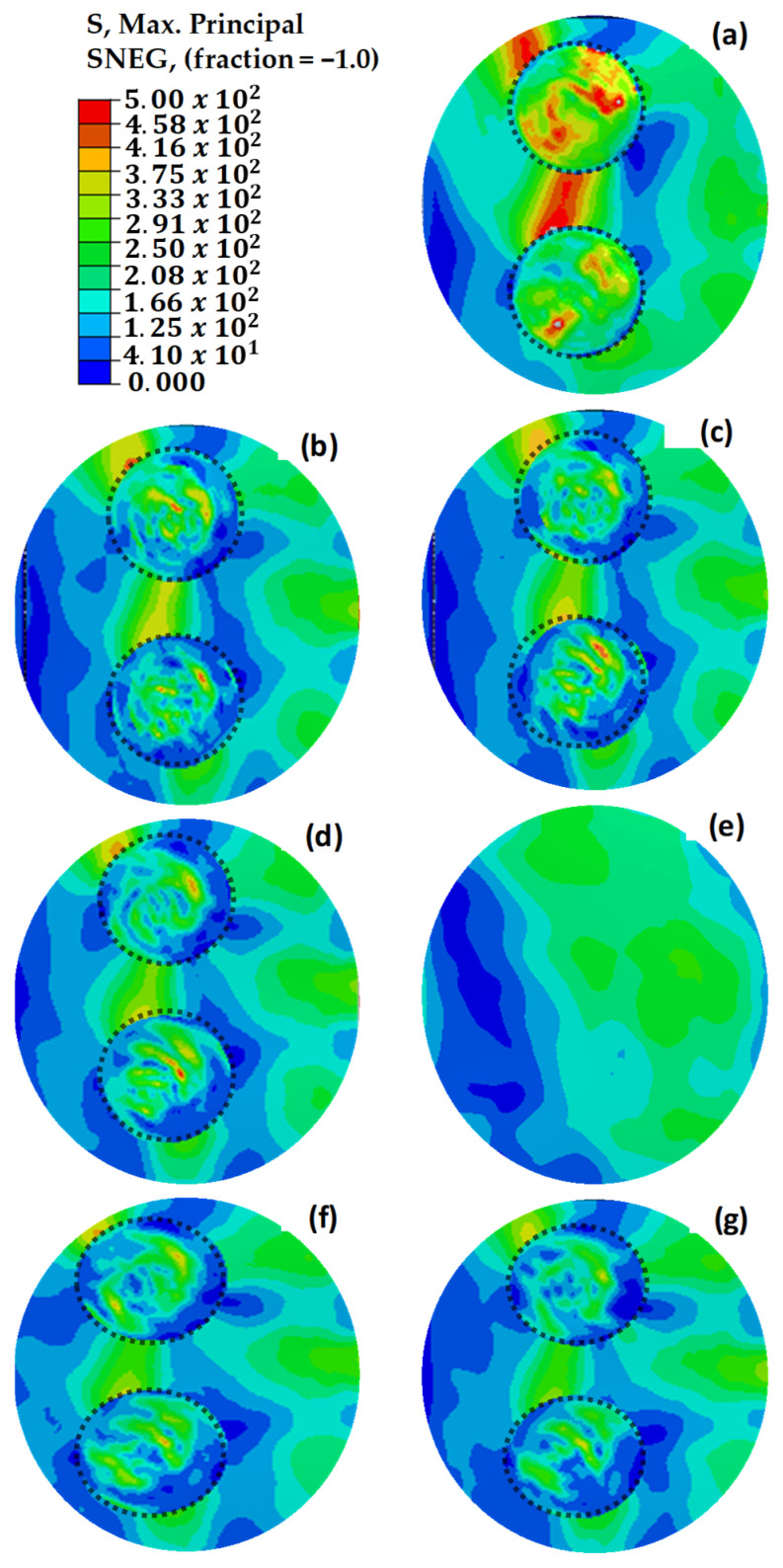
Maximum principal stress (MPa) distributions of repaired, pristine, and damaged structures. (**a**) The double main structure with 3 missing layers. (**b**) The double main structure with 2 missing layers. (**c**) The double main structure with 1 missing layer. (**d**) The repair of the double structure without extra layers. (**e**) The pristine main structure. (**f**) The repaired structure with one extra 45° layer. (**g**) The repaired structure with two extra 0–45° layers. The dotted circles indicate the boundaries of the repaired regions.

**Figure 24:**
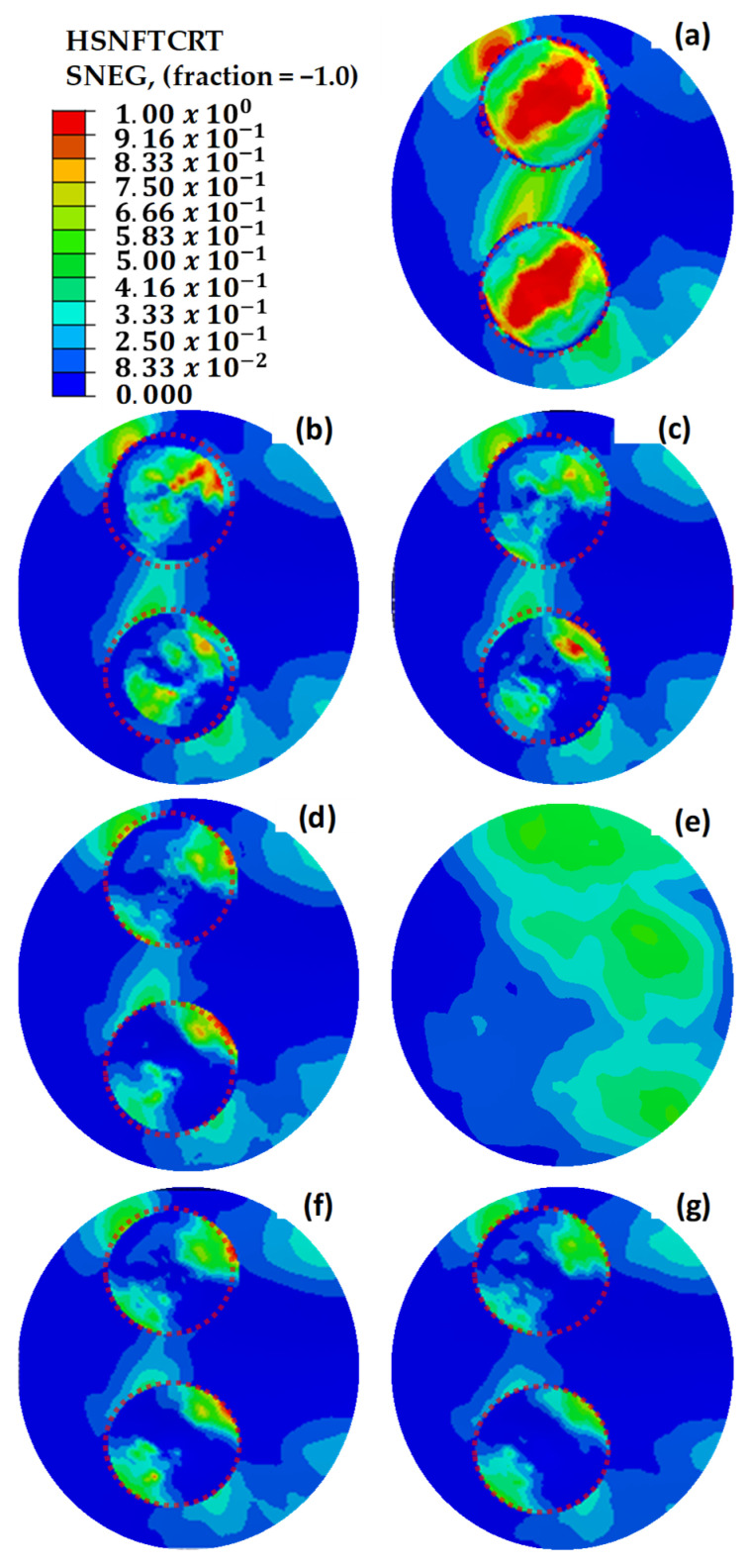
Hashin damage initiation values of repaired, pristine, and damaged structures. (**a**) The double main structure with 3 missing layers. (**b**) The double main structure with 2 missing layers. (**c**) The double main structure with 1 missing layer. (**d**) The repair of the double structure without extra layers. (**e**) The pristine main structure. (**f**) The repaired structure with one extra 45° layer. (**g**) The repaired structure with two extra 0–45° layers. The dotted circles indicate the boundaries of the repaired regions.

The dotted circles in Figure 22, Figure 23 and Figure 24 indicate the boundaries of the repaired regions. Figure 22 illustrates the maximum principal strain values for repaired, pristine, and damaged structures, where two independent repairs were applied in Section 1. Strain values exceeding 5000 με were observed in Figure 22a–d, leading to material failure. However, the addition of extra layers effectively reduced the strain values below the maximum allowable limit of 5000 με. Notably, along the beam line, high stress and strain concentrations were identified between the two circular repair regions on the load path. Extending the additional layers by 12.5 mm mitigated the discontinuities in these high-stress regions, resulting in a significant reduction in strain values.

Figure 23 shows the maximum principal stress values for the double-region repaired structures, demonstrating alignment with the strain distribution observed in Figure 22. The results confirm that the critical stress regions correspond to the identified high-strain zones.

Figure 24 depicts the Hashin damage initiation values for repaired, pristine, and damaged structures. Breakages were evident in Figure 24a–d, which is consistent with the strain and stress analysis results. The findings from the Hashin model validate these analyses, as they highlight critical areas of damage and confirm the effectiveness of the repairs in reducing high-strain concentrations.

## 4. Conclusions

This study investigates the mechanical performance of stepped-lap repairs applied to a full-scale composite aircraft wing under realistic loading conditions. The simulations were first validated by performing experimental tensile tests on repaired specimens, revealing critical insights into repair strategies for carbon fiber-reinforced plastic (CFRP) structures. Then, CFD analyses are performed to determine the aerodynamic forces acting on the full-scale wing model. Realistic boundary conditions are applied to the model, including the aerodynamic pressures and the weight of fuel and ammunition. The most critical region is determined on the wing by investigating the stress levels. After completing these steps, various damage and repair scenarios are virtually performed on the critical region of the wing. Two distinct approaches are primarily investigated: the repairs applied to a single damaged region and the repairs addressing two closely located damaged regions.

For single-region repairs, applying up to two additional layers significantly enhances structural strength, as evidenced by reduced tensile strain. However, adding a third layer leads to higher strain values, diminishing repair effectiveness. In scenarios involving two closely located damaged regions, the mechanical performance improves with additional layers, but the gains are less pronounced compared to single-region repairs. The analysis further highlights the importance of repair geometry, with circular repairs outperforming square repairs by minimizing stress concentrations and ensuring a more uniform strain distribution.

In transitioning from coupon-level specimens to the full-scale aircraft wing model, the distribution of damage and stress exhibits a more widespread character compared to the localized coupon failure. This behavior is attributed to the complex interaction between the wing’s internal structural layout and the realistic service loads. Unlike the isolated coupons, the full-scale model accounts for the presence of internal components and the pressurized environment of fuel-carrying segments.

In a global wing structure, the skin-patch assembly is subjected to combined biaxial and shear loading, which can trigger localized out-of-plane deformations and pre-buckling behavior. However, the internal ribs and spars, coupled with the stabilizing effect of the fuel−structure interaction, act as a corrective constraint against excessive buckling. This constraint redistributes the energy that would otherwise cause localized catastrophic failure in a coupon. Consequently, the “out-of-plane” behavior associated with secondary bending is dispersed across a larger area in the full-scale model. This results in the “widespread” damage patterns observed in the wing skin analysis, providing a more realistic representation of how a structural-level repair manages load paths compared to the concentrated stress states found in simplified coupon testing.

This explains the more widespread stress distribution in the full-scale model compared to the hyper-localized failure in coupons. The energy that causes localized failure in a coupon is redistributed across a larger area in the wing skin due to these global structural constraints, preventing the premature “peeling” failure and allowing the patch to carry higher loads before total structural compromise.

These findings emphasized the need for careful optimization of repair strategies, including layer configurations and geometric considerations, to achieve maximum structural integrity. The methodologies and results provided valuable guidance for advancing maintenance practices in CFRP-based aerospace structures and addressing the challenges of large-scale composite repairs. Future research could explore the impact of dynamic loading conditions and material aging on repair performance to further refine these strategies. The findings provided a deeper insight in order to reveal the effects of repair methods using computational modeling techniques.

## Figures and Tables

**Table 1:** Material properties of the M21/AS4C [38] and Hexforce G0904 using HYSOL EA 9396 adhesive.

Property	Symbol	Hexforce G0904	M21/AS4C
Elastic Modulus (GPa)	*E*_11_	49.6	61.0
	*E*_22_	49.6	61.0
	*E*_33_	8.0	8.9
Shear Modulus (GPa)	*G*_12_	3.3	4.2
	*G*_13_, *G*_23_	2.8	3.8
Tensile Strength (MPa)	*X*_T_	517	930
	*Y*_T_	517	940
Compression Strength (MPa)	*X*_C_	483	883
	*Y*_C_	483	883
Shear Strength (MPa)	*S*_12_	60	96
	*S*_13_, *S*_23_	34	64
Poisson ratio	*ν*_12_	0.045	0.05
	*ν*_13_, *ν*_23_	0.28	0.3

**Table 2:** Mechanical properties of the FM-300 K and HYSOL EA 9396 adhesive.

Property	Symbol	FM-300K	HYSOL EA 9396
Elastic Modulus (GPa)	*E*	3.12	2.7
Shear Modulus (GPa)	*G*	0.9	0.7
Tensile Strength (MPa)	$t_{n}^{0}$	72	55
Shear Strength (MPa)	$t_{s}^{0}$ $,\;t_{t}^{0}$	42	26
Tensile Stiffness (N/mm^3^)	*K*_n_	15,600	10^6^
Shear Stiffness (N/mm^3^)	*K*_s_, *K*_t_	4500	10^6^
Mode-1 Fracture Energy (N/mm)	*G*_IC_	1.1	0.3
Mode-2,3 Fracture Energy (N/mm)	*G*_IIC_, *G*_IIIC_	4.8	0.5

**Table 3:** Mechanical properties of the core.

Property	Symbol	Core
Elastic Modulus (MPa)	*E*_11_	120
	*E*_22_	110
	*E*_33_	-
Shear Modulus (GPa)	*G*_12_	32
Tensile Strength (MPa)	*X*_T_	1.65
	*Y*_T_	1.65
Shear Strength (MPa)	*S*_12_	1.4
Poisson ration	*ν*_12_	0.25

**Table 4:** The scenarios of the pristine, damaged, and stepped-lap repaired specimens for single-region cases.

Scenario Number	Type of Analysis	Repair Geometry	Damaged Ply Number	Number and Orientation of Extra Layers	Representation of Repair 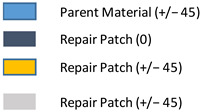
1	Damaged Structure	Circular	3	−	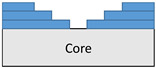
2	Damaged Structure	Circular	2	−	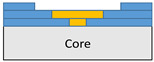
3	Damaged Structure	Circular	1	−	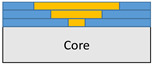
4	Repaired Structure	Circular	0	−	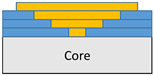
5	Parent Structure	−	0	−	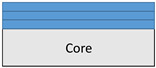
6	Repaired Structure	Circular	0	1 (+/−45)	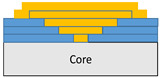
7	Repaired Structure	Circular	0	2 (+/−45)	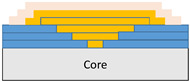
8	Repaired Structure	Circular	0	2 (0/45)	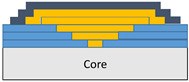
9	Repaired Structure	Square	0	1 (+/−45)	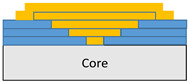
10	Repaired Structure	Square	0	2 (0/45)	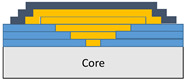

**Table 5:** The scenarios of the pristine, double-damaged, and stepped-lap repaired specimens for the double repair cases.

Scenario Number	Type of Analysis	Repair Geometry	Damaged Ply Number	Number and Orientation of Extra Layers	Representation of Repair 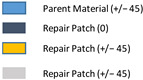
11	Double Damaged	Circular	3	−	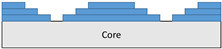
12	Double Damaged	Circular	2	−	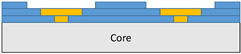
13	Double Damaged	Circular	1	−	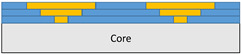
14	Double Repaired	Circular	0	−	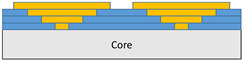
15	Parent Structure	−	0	−	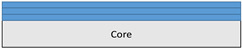
16	Double Repaired	Circular	0	1 (+/−45)	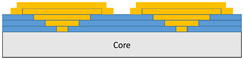
17	Double Repaired	Circular	0	2 (0/45)	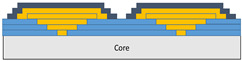

**Table 6:** Tensile test results of pristine and stepped-lap specimens.

Type of Specimen	Method	Average Tensile Stress (MPa)	Average Strain (με)	Coefficient of Variation (%)	Repair Improvement Ratio
Pristine specimen	Test	508.25	10,601	2.25	-
7-Layer Repair	Test	362.4	7518	7.3	71.3
9-Layer Repair	Test	356.4	7385	8.4	70.1
Pristine specimen	Numerical	491.4	10,231	-	-
7-Layer Repair	Numerical	370.8	7695	-	73
9-Layer Repair	Numerical	338	7011	-	66.53

**Table 7:** The maximum tensile strain values of the pristine, damaged, and stepped-lap repaired specimens given in Table 4.

Scenario Number	Maximum Tensile Strain (με)	Representation of Repair 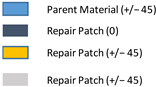
1	6700	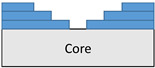
2	3436	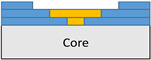
3	2754	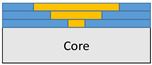
4	2628	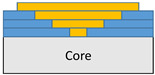
5	2631	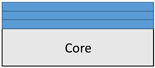
6	2560	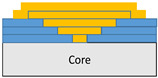
7	3146	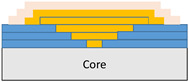
8	3626	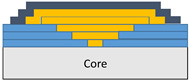
9	4592	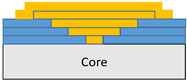
10	4337	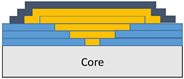

**Table 8:** The maximum principal tensile strain values of the pristine, double-damaged, and stepped-lap repaired specimens given in Table 5.

Scenario Number	Maximum Tensile Strain (με)	Representation of Repair 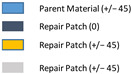
11	8105	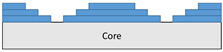
12	6016	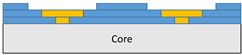
13	5501	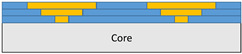
14	5031	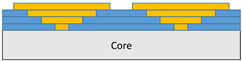
15	2631	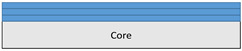
16	4673	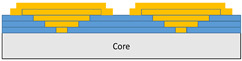
17	4539	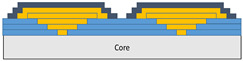

## Data Availability

Data is contained within the article.
